# A Dangerous Drug

**Published:** 1890-02

**Authors:** St. James Gazette


### A DANGEROUS DRUG.

Cocaine, as is now well known, is a very valuable but an exceedingly dangerous drug, and M. Dufournier has lately published in the *Archives de Medecine* the remarkable results of his investigations into its use. Cases where accidents have occurred are very numerous, and there is hardly a surgeon using cocaine who has not had occasion to witness them. As early as in 1887, Dr. Mattison published the account of forty such instances, and the roll of victims who have lost their life from a dose of cocaine has now reached as high as nine. In a large number of cases it has given rise to a species of poisoning, from which the patient usually recovers. Among the phenomena characterizing this form of poisoning, one instance observed in a patient of Dr. E. Bradley is worthy of mention. This patient was taken with facial paralysis, from which he did not recover for six months. Other symptoms are hallucinations, great excitement and cerebral agitation, and, finally, Dr. Leslie Callog-han in one case saw the entire body covered by a scarletiniform rash.

Dr. Szunman, wishing to remove a large wart situated at the base of the thumb of a young girl of twenty, injected under the skin, close to the wart, one cubic centimeter of a one-in-ten solution of cocaine. The patient felt no pain, but as the little wound was being sewed together, she suddenly lost color and fainted, her pulse became weak and slow, and her hands and feet stiffened. Water was dashed in her face and she recovered consciousness, but she did not regain at once her sense of feeling, as she kept asking where her hands were. By this time the stiffening had extended to the whole of her person, but these alarming symptoms quieted down little by little, and by half an hour's time they all came to a happy end. This case represents the mildest form of cocaine poisoning. Between this form and the cases in which death ensued come in a series of severer forms, in which the alarming symptoms lasted three hours to five or six days.—*St. James Gazette.*

